# Supplementary material for: Vascular FLRT2 regulates venous-mediated angiogenic expansion and CNS barriergenesis
Source: Nat Commun. 2024 Nov 29;15:10372. doi: 10.1038/s41467-024-54570-x (PMC11604978; doi:10.1038/s41467-024-54570-x)
Supplement: Supplementary file 1 — Supplementary Information [file 41467_2024_54570_MOESM1_ESM.pdf]

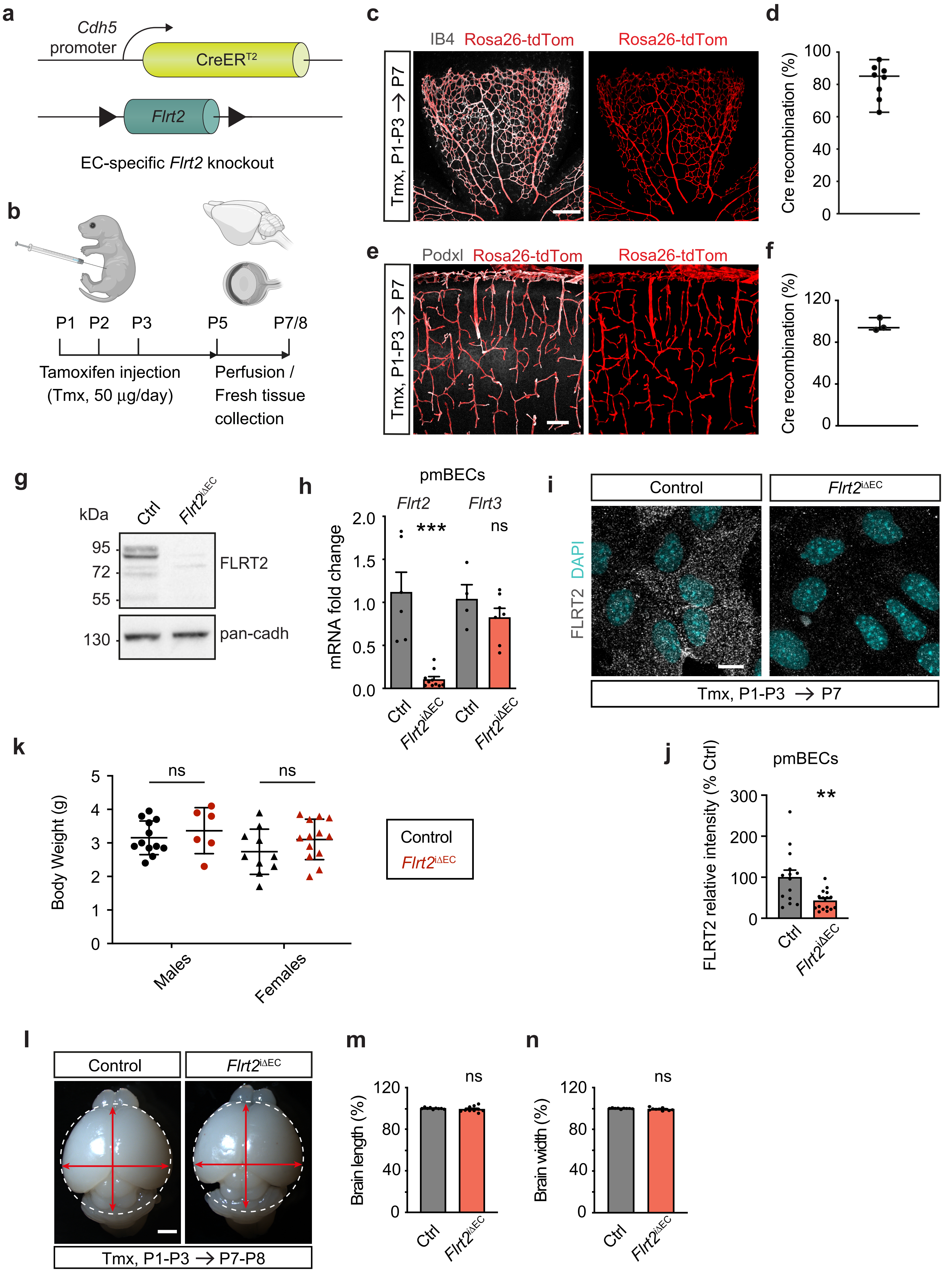

a
Cdh5 promoter
CreER^{T2}
Flrt2
EC-specific Flrt2 knockout
b
P1 P2 P3 P5 P7/8
Tamoxifen injection (Tmx, 50 µg/day)
Perfusion / Fresh tissue collection
c
IB4 Rosa26-tdTom
Rosa26-tdTom
Tmx, P1-P3 → P7
d
Cre recombination (%)
e
Podxl Rosa26-tdTom
Rosa26-tdTom
Tmx, P1-P3 → P7
f
Cre recombination (%)
g
kDa
Ctrl
Flrt2^{iΔEC}
FLRT2
pan-cadh
h
pmBECs
Flrt2 Flrt3
***
ns
mRNA fold change
i
Control
Flrt2^{iΔEC}
FLRT2 DAPI
Tmx, P1-P3 → P7
j
pmBECs
**
FLRT2 relative intensity (% Ctrl)
k
Body Weight (g)
ns
Males
Females
Control
Flrt2^{iΔEC}
l
Control
Flrt2^{iΔEC}
Tmx, P1-P3 → P7-P8
m
Brain length (%)
ns
n
Brain width (%)
ns

Supplementary Fig. 1| FLRT2 EC-specific deletion.

(a) Schematic representation of *Flrt2* endothelial specific knockout strategy in Cdh5(PAC)-CreERT2:Flrt2$^{lox/lox}$ (*Flrt2*$^{i\Delta EC}$) mice. **(b)** Experimental design of *Flrt2* gene deletion by Cre recombination. 4-hydroxytamoxifen (Tmx) was applied from P1 to 3 and tissue collected at P5 and P7-8. Cartoon created in Biorender.com (Kirchmaier, B. (2024) BioRender.com/ u51i186). **(c, e)** Flat-mounted retina (c) and neocortical brain slices (e) from P7 Cdh5-CreERT2:Rosa26tdTomato mice injected from P1 to P3 with Tmx. Cells undergoing Cre-mediated recombination expressed the fluorescent protein tdTomato, used to assess specificity and efficiency in blood vessels, co-stained with IB4 (c) or podocalyxin (Podxl) (e). **(d, f)** Percentage of tdTomato-positive signal per IB4-positive (d) and Podxl-positive signal (f). n = 8 mice (d), 3 mice (f). Horizontal bars show the median value. **(g)** Immunoblot showing FLRT2 protein levels in primary lung ECs isolated from P8 control and *Flrt2*$^{i\Delta EC}$ mice. Pan-cadherin (pan-cadh) was used as loading control. **(h)** *Flrt2* and *Flrt3* mRNA levels from primary mouse brain ECs (pmBECs) isolated from P5 control and *Flrt2*$^{i\Delta EC}$ mice. n = 6 control, 10 mutant mice (*Flrt2*); 4 control, 7 mutant mice (*Flrt3*). Two-tailed Mann-Whitney test, p = 0.0002 (*Flrt2*), two-tailed unpaired t-test, p = 0.282 (*Flrt3*). **(i)** pmBECs isolated from P7 control and *Flrt2*$^{i\Delta EC}$ mice stained for FLRT2 and DAPI. **(j)** FLRT2 fluorescence intensity quantification in control and *Flrt2*$^{i\Delta EC}$ pmBECs. n = 14 control, 16 *Flrt2* mutant pictures from 2 mice per genotype, one litter. Two-tailed unpaired t-test, p = 0.003. **(k)** Body weight of control and *Flrt2*$^{i\Delta EC}$ male and female mice at P7-8. n = 12 control males, 6 mutant males, 10 control females, 13 mutant females. Two-tailed unpaired t-test, p = 0.464 (males), p = 0.185 (females). **(l)** Example of whole control and *Flrt2*$^{i\Delta EC}$ brains. **(m, n)** Quantifications of brain length (m) and width (n) of control and *Flrt2*$^{i\Delta EC}$ mice. n = 9 control, 10 mutant mice. Two-tailed unpaired t-test, p = 0.407 (length), p = 0.125 (width).

Scale bars: 200 µm (c), 100 µm (e), 10 µm (i), 1 mm (l). Data are shown as mean ± SEM. **P < 0.01, ***P > 0.001, ns= not significant.

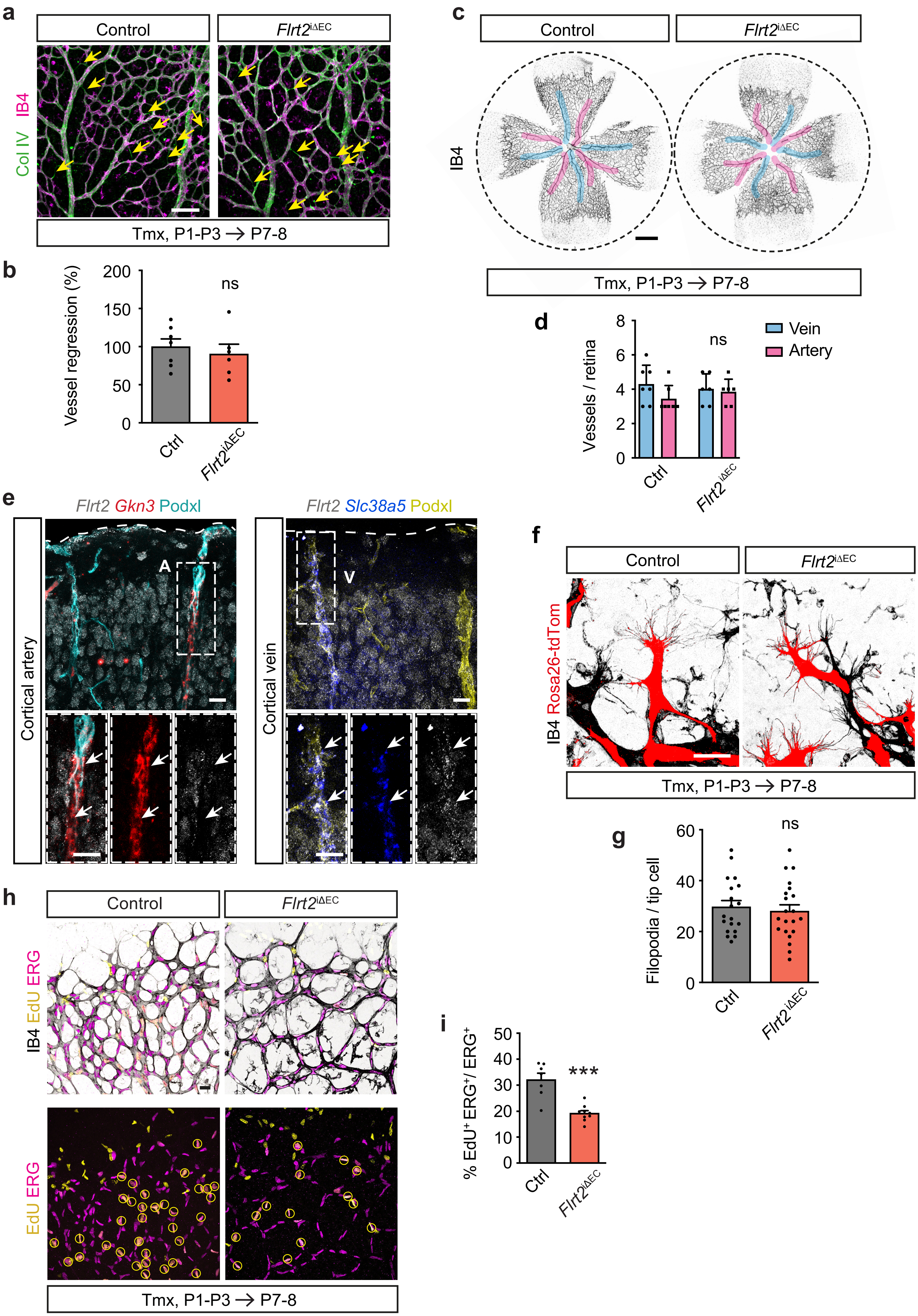
a
Control
Flrt2iΔEC
Col IV IB4
Tmx, P1-P3 → P7-8
b
Vessel regression (%)
200
150
100
50
0
ns
Ctrl
Flrt2iΔEC
c
Control
Flrt2iΔEC
IB4
Tmx, P1-P3 → P7-8
d
Vessels / retina
8
6
4
2
0
ns
Vein
Artery
Ctrl
Flrt2iΔEC
e
Flrt2 Gkn3 Podxl
Flrt2 Slc38a5 Podxl
Cortical artery
Cortical vein
A
V
f
Control
Flrt2iΔEC
IB4 Rosa26-tdTom
Tmx, P1-P3 → P7-8
g
Filopodia / tip cell
60
40
20
0
ns
Ctrl
Flrt2iΔEC
h
Control
Flrt2iΔEC
IB4 EdU ERG
EdU ERG
Tmx, P1-P3 → P7-8
i
% EdU+ ERG+/ ERG+
50
40
30
20
10
0
***
Ctrl
Flrt2iΔEC

Supplementary Fig. 2| Characterization of vascular parameters in FLRT2 deficient retinas.

(a) Flat mounted retinas stained for Collagen IV (Col IV) and IB4 to visualize blood vessel regression**.** Col IV^{+} IB4^{-} empty sleeves are marked with yellow arrows. **(b)** Quantification of vessel regression as Col IV^{+} IB4^{-} empty sleeves per imaging field. n = 7 control, 6 mutant mice. Two-tailed unpaired t-test, p = 0.562. **(c)** Representative images of retinas from control and *Flrt2*$^{i\Delta EC}$ P7-P8 littermates stained with IB4. Arteries (pink) and veins (blue) are highlighted. **(d)** Quantification of the total number of veins and arteries per retina in mutant and control mice at P7-8. n = 7 control, 6 mutant mice. Two-way ANOVA, Alpha = 0.050. **(e)** Negative *Flrt2* (grey) expression in a cortical artery (A) expressing the *Gkn3* marker (red) and positive *Flrt2* signal in a vein (V) expressing *Scl38a5* (blue). Podxl immunostaining is used as a general marker of the vasculature. **(f)** Recombinant tip cells in the retinal vascular front of Cdh5-CreERT2:Rosa26tdTomato (control) and *Flrt2*$^{i\Delta EC}$:Rosa26tdTomato P7-P8 mice. Upon Cre-mediated recombination ECs express tdTomato reporter protein (depicted as red). **(g)** Quantification of the number of filopodia per tip cell. n =19 control, 21 mutant cells from 4 animals per genotype. Two-tailed unpaired t-test, p = 0.632. **(h)** Retinal vascular fronts from control and *Flrt2*$^{i\Delta EC}$ P7-P8 mice with proliferative cells labelled with EdU, EC nuclei stained with ERG, and blood vessels with IB4. **(i)** Quantification of % EdU^{+}ERG^{+} cells in total ERG^{+} cells per image. n = 7 control, 9 mutant animals. Two-tailed unpaired t-test, p = 0.0002. Scale bars: 100 µm (a), 500 µm (c), 25 µm (e, f, h). Data are shown as mean ± SEM. ***P > 0.001, ns = not significant.

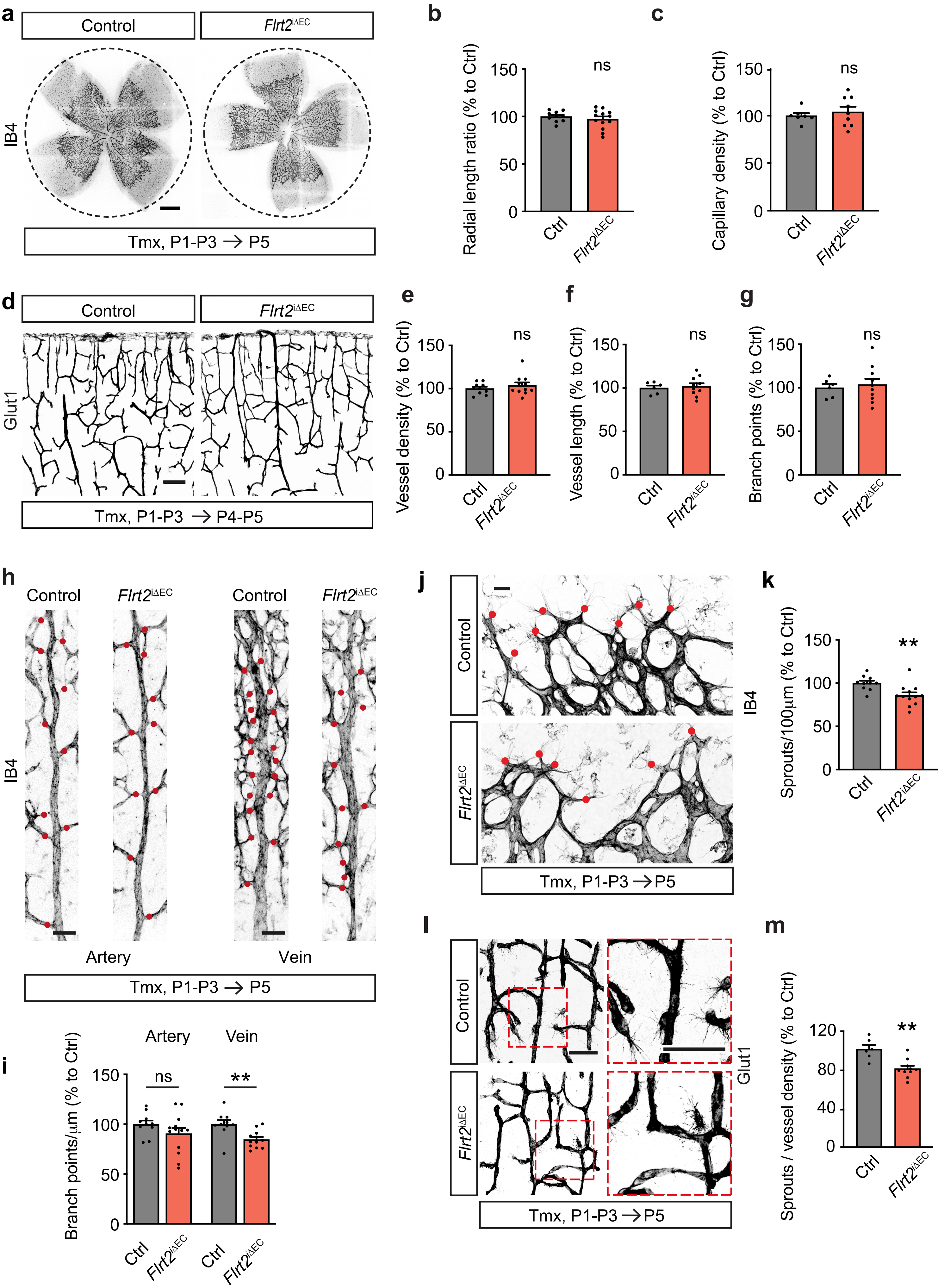

a
Control
Flrt2iΔEC
IB4
Tmx, P1-P3 → P5
b
Radial length ratio (% to Ctrl)
ns
150
100
50
0
Ctrl
Flrt2iΔEC
c
Capillary density (% to Ctrl)
ns
d
Control
Flrt2iΔEC
Glut1
Tmx, P1-P3 → P4-P5
e
Vessel density (% to Ctrl)
f
Vessel length (% to Ctrl)
g
Branch points (% to Ctrl)
h
Control
Flrt2iΔEC
IB4
Artery
Vein
Tmx, P1-P3 → P5
i
Artery
Vein
Branch points/µm (% to Ctrl)
**
j
Control
Flrt2iΔEC
Tmx, P1-P3 → P5
IB4
k
Sprouts/100µm (% to Ctrl)
l
Control
Flrt2iΔEC
Glut1
Tmx, P1-P3 → P5
m
Sprouts / vessel density (% to Ctrl)
120
80
40

Supplementary Fig. 3| Sprouting defects precede the decreased vascularization in *Flrt2* mutants.

(a) P5 flat-mounted retinas from control and *Flrt2*$^{i\Delta EC}$ littermates injected with 4-hydroxytamoxifen (Tmx) from P1 to P3, stained with IB4 to visualize blood vessels. **(b**, **c)** Quantification of the vascular radial growth ratio (b), and the capillary density (c) in P5 control and mutant retinas. n = 10 control and 13 mutant animals (b), and 7 control and 9 mutant animals (c). Two-tailed unpaired t-test, p = 0.457 (b), 0.534 (c). **(d)** Glut1 staining of control and *Flrt2*$^{i\Delta EC}$ cortices at P4-P5 after Tmx administration from P1 to P3. **(e - g)** Quantification of vessel density (e), vessel length (f) and number of branch points (g) in mouse cerebral cortices. n = 10 control, 11 mutant (e), 6 control and 10 mutant mice (f, g). Two-tailed unpaired t-test, p = 0.383 (e), 0.715 (f), 0.704 (g). **(h)** Representative images of arteries and veins from control and *Flrt2*$^{i\Delta EC}$ retinas stained with IB4 at P5. Red dots indicate branch points from the mother vessel. **(i)** Quantification of the number of branch points in main arteries (left) and veins (right) per vessel length. n = 10 control and 13 mutant (arteries), 10 control and 12 mutant (veins) mice. Two-tailed unpaired t-test, p = 0.205 (arteries), 0.003 (veins). **(j)** P5 retinal vascular fronts stained with IB4. Red dots indicate cellular protrusions identified as angiogenic sprouts. **(k)** Quantification of the number of sprouts per 100 μm of vascular front. n = 10 control and 12 mutant mice. Two-tailed unpaired t-test, p = 0.006. (**l**) Control and *Flrt2*$^{i\Delta EC}$ cerebral cortices at P5 stained for Glut1 to visualize vessel sprouts. (**m**) Quantification of the number of sprouts per vessel density at P5 in *Flrt2*$^{i\Delta EC}$ and control littermates. n = 6 control and 10 animals per genotype. Two-tailed unpaired t-test, p = 0.002.

Scale bars: 500 μm (a), 100 μm (d), 50 μm (h, j, l). Data are shown as mean ± SEM. **P < 0.01, ns = not significant.

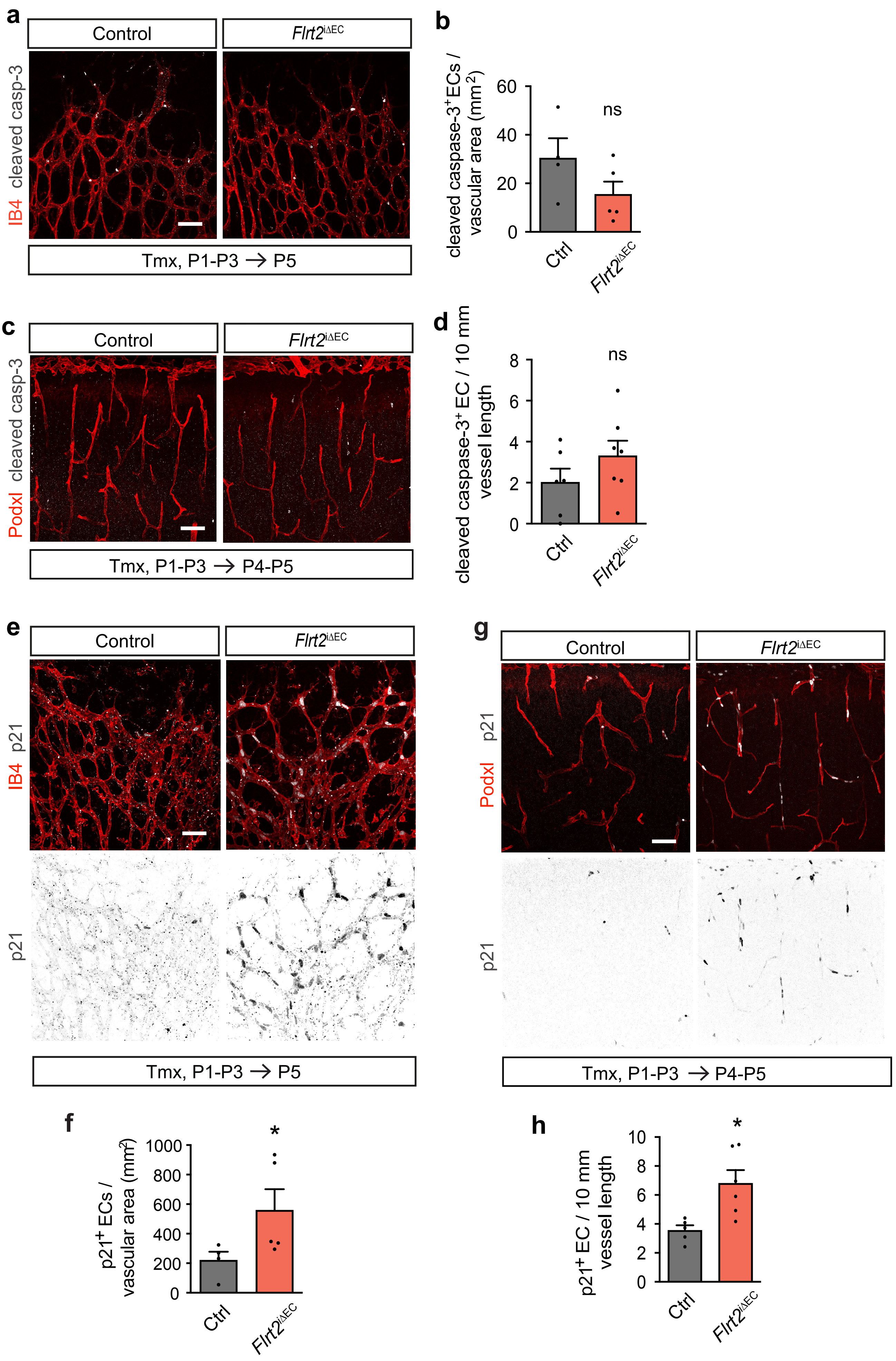

a
Control
Flrt2iΔEC
IB4 cleaved casp-3
Tmx, P1-P3 → P5
b
cleaved caspase-3+ECs / vascular area (mm2)
60
40
20
0
ns
Ctrl
Flrt2iΔEC
c
Control
Flrt2iΔEC
Podxl cleaved casp-3
Tmx, P1-P3 → P4-P5
d
cleaved caspase-3+ EC / 10 mm vessel length
8
6
4
2
0
ns
Ctrl
Flrt2iΔEC
e
Control
Flrt2iΔEC
IB4 p21
p21
Tmx, P1-P3 → P5
g
Control
Flrt2iΔEC
Podxl p21
p21
Tmx, P1-P3 → P4-P5
f
p21+ ECs / vascular area (mm2)
1000
800
600
400
200
0
*
Ctrl
Flrt2iΔEC
h
p21+ EC / 10 mm vessel length
10
8
6
4
2
0
*
Ctrl
Flrt2iΔEC

Supplementary Fig. 4| FLRT2 deficiency increases EC cell cycle arrest but not EC death.

(a, c) Flat mounted retinas (a) and cortical brain slices (c) stained for apoptosis marker cleaved caspase-3 (cleaved casp-3) and blood vessels (IB4 or Podxl). **(b, d)** Quantification of cleaved caspase-3^{+} ECs in control and *Flrt2*$^{i\Delta EC}$ P5 retinas (b) and P4-P5 brain neocortices (d). n = 4 control and 5 mutant mice (b), 6 control and 7 mutant mice (d). Two-tailed unpaired t-test, p = 0.155 (b), 0.224 (d). **(e, g)** Flat mounted retinas (e) and cortical brain slices (g) stained for cell cycle arrest marker p21 and blood vessels (IB4 or Podxl). **(f, h)** Quantification of $p21^{+}$ ECs in control and *Flrt2*$^{i\Delta EC}$ P5 retinas (f) and P4-P5 brain neocortices (h). n = 4 control and 5 mutant mice (f), 5 control and 6 mutant mice (h). Two-tailed Mann-Whitney test, p = 0.032 (f), two-tailed unpaired t-test, p = 0.014 (h).

Scale bars: 50 µm. Data are shown as mean ± SEM. *$P < 0.05$, ns = not significant.

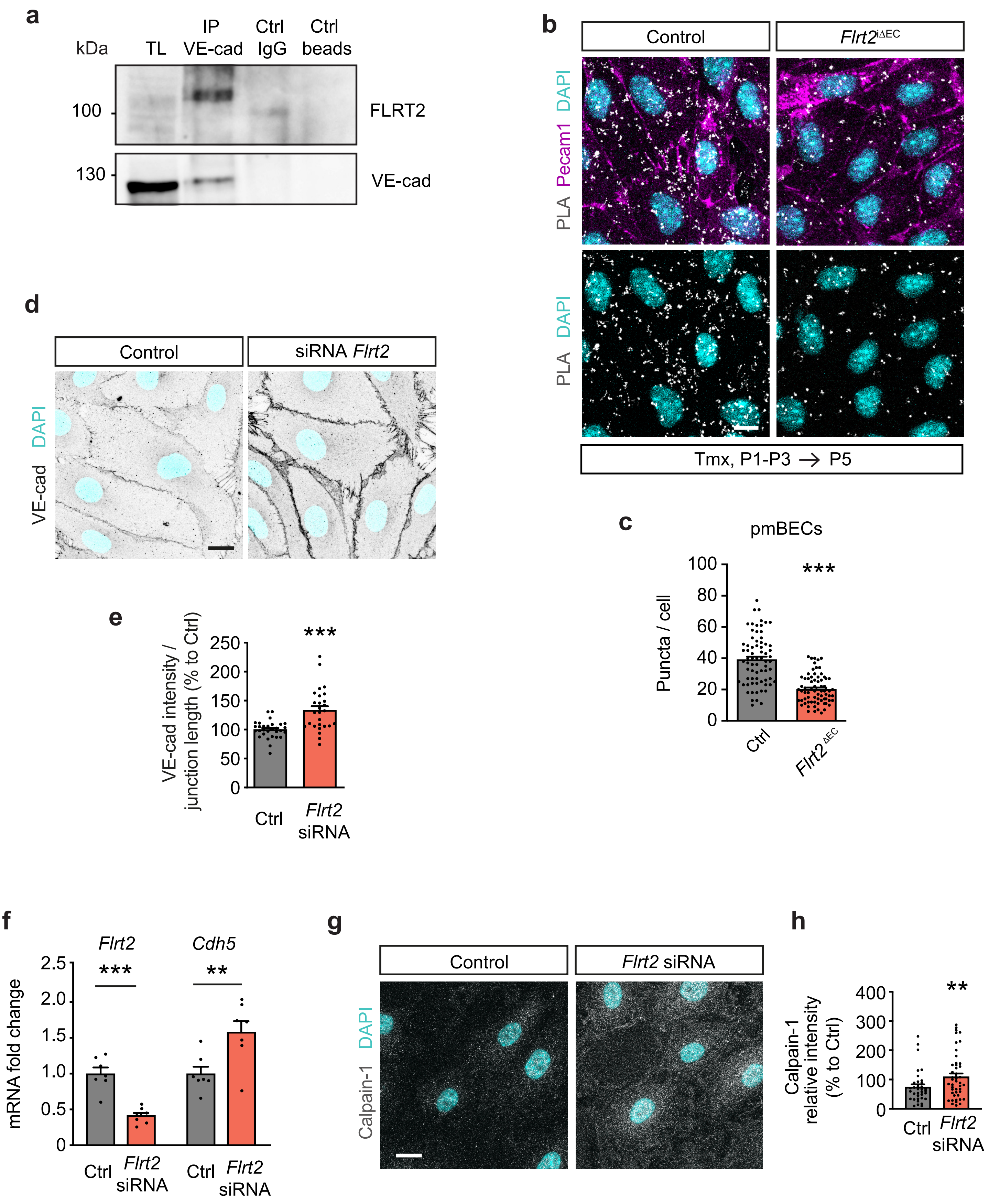
a
kDa
TL
IP
VE-cad
Ctrl
IgG
Ctrl
beads
100
FLRT2
130
VE-cad
b
Control
Flrt2iΔEC
PLA Pecam1 DAPI
PLA DAPI
Tmx, P1-P3 → P5
c
pmBECs
Puncta / cell
100
80
60
40
20
0
***
Ctrl
Flrt2ΔEC
d
Control
siRNA Flrt2
VE-cad DAPI
e
VE-cad intensity / junction length (% to Ctrl)
250
200
150
100
50
0
***
Ctrl
Flrt2 siRNA
f
Flrt2
Cdh5
mRNA fold change
2.5
2.0
1.5
1.0
0.5
0
***
**
Ctrl
Flrt2 siRNA
Ctrl
Flrt2 siRNA
g
Control
Flrt2 siRNA
Calpain-1 DAPI
h
Calpain-1 relative intensity (% to Ctrl)
400
300
200
100
0
**
Ctrl
Flrt2 siRNA

Supplementary Fig. 5| FLRT2 modulates the organization of adherens junctions in the CNS.

(a) Immunoprecipitation of VE-cadherin and immunodetection of FLRT2 and VE-cadherin from HUVEC cultures. **(b)** PLA in primary mouse brain EC (pmBECs) cultures. White puncta indicate FLRT2 and VE-cadherin being in close proximity (< 40 nm). Cells were stained with Pecam1 and DAPI to visualize EC junctions and nuclei, respectively. Note the major presence of PLA puncta along the Pecam1^{+} junctions in the cells isolated form control mice, indicating that FLRT2 and VE-cadherin interact at the cell surface. **(c)** PLA signal quantification as puncta per cell. n = 71 control and 72 mutant cells from 2 animals per genotype. Two-tailed Mann-Whitney test, p < 0.0001. **(d)** HUVEC treated with control and *Flrt2*-specific siRNAs immunostained for VE-cadherin and DAPI. **(e)** Quantification of VE-cadherin intensity per cellular junction length. n = 30 control and 27 *Flrt2* siRNA-treated images per condition, from 3 independent experiments. Two-tailed unpaired t-test, p <0.0001. **(f)** mRNA expression of *Flrt2* and *Cdh5* in HUVEC transfected with control and *Flrt2*-specific siRNAs. n = 7 independent experiments. Two-tailed t-test, p < 0.0001 (*Flrt2*), 0.01 (*Cdh5*). **(g)** HUVEC cultures transfected with control and *Flrt2*-specific siRNAs stained for Calpain-1 and DAPI. **(h)** Quantification of the fluorescence intensity of Calpain-1 staining per cell. n = 33 control and 46 *Flrt2* siRNA-treated cells, 1 representative experiment from 3 independents experiments. Two-tailed Mann-Whitney test, p = 0.008.

Scale bars: 10 μm (b), 15 μm (d), 20 μm (g). Data are shown as mean ± SEM. **P < 0.01, ***P < 0.001.

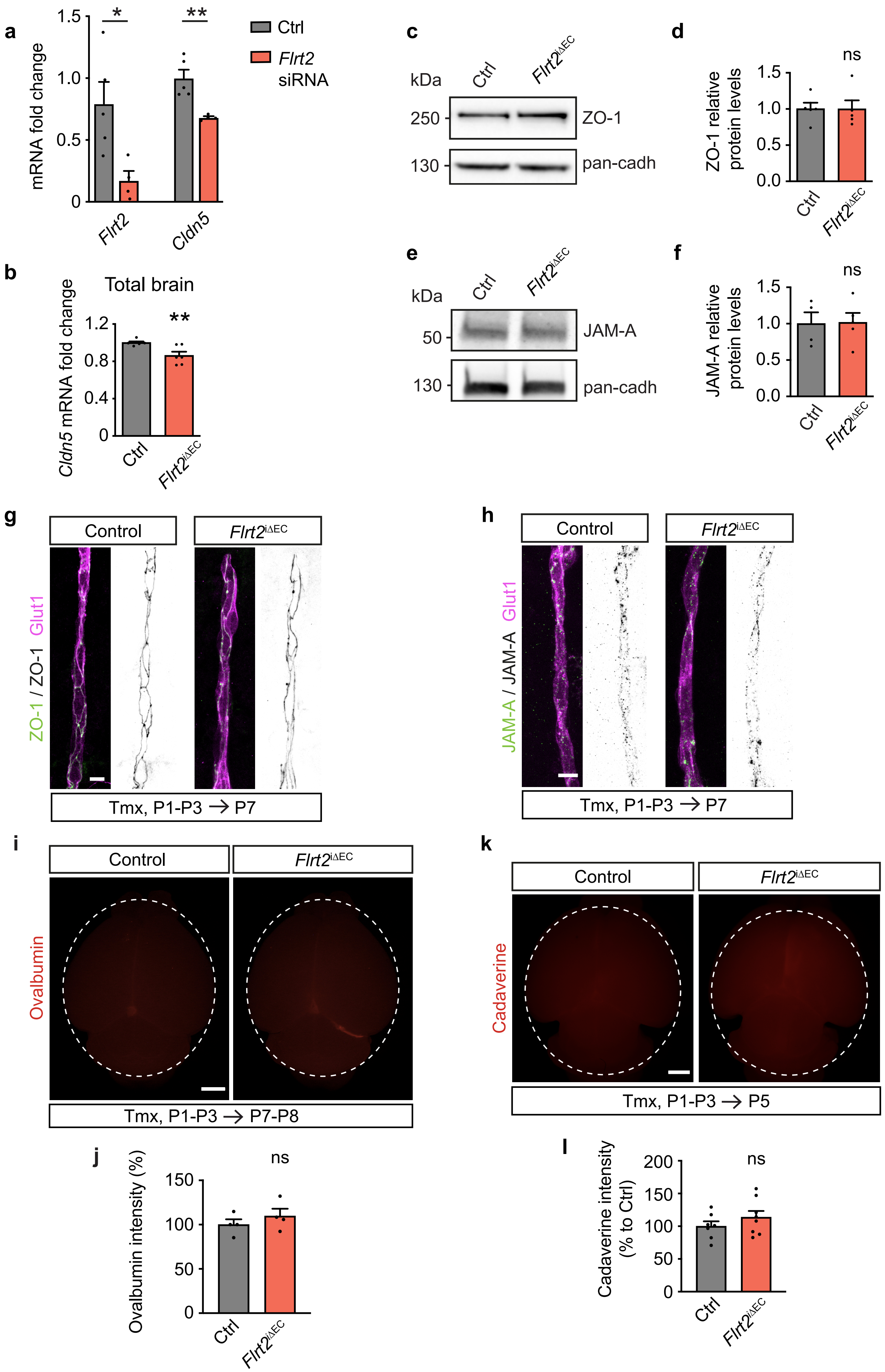

a
mRNA fold change
1.5
1.0
0.5
0
*
**
Ctrl
Flrt2 siRNA
Flrt2
Cldn5
b
Total brain
Cldn5 mRNA fold change
1.2
0.8
0.4
0
**
Ctrl
Flrt2iΔEC
c
kDa
Ctrl
Flrt2iΔEC
250
130
ZO-1
pan-cadh
d
ZO-1 relative protein levels
ns
1.5
1.0
0.5
0.0
Ctrl
Flrt2iΔEC
e
kDa
Ctrl
Flrt2iΔEC
50
130
JAM-A
pan-cadh
f
JAM-A relative protein levels
ns
1.5
1.0
0.5
0.0
Ctrl
Flrt2iΔEC
g
Control
Flrt2iΔEC
ZO-1 / ZO-1 Glut1
Tmx, P1-P3 → P7
h
Control
Flrt2iΔEC
JAM-A / JAM-A Glut1
Tmx, P1-P3 → P7
i
Control
Flrt2iΔEC
Ovalbumin
Tmx, P1-P3 → P7-P8
k
Control
Flrt2iΔEC
Cadaverine
Tmx, P1-P3 → P5
j
Ovalbumin intensity (%)
ns
150
100
50
0
Ctrl
Flrt2iΔEC
l
Cadaverine intensity (% to Ctrl)
ns
200
150
100
50
0
Ctrl
Flrt2iΔEC

Supplementary Fig. 6| Endothelial FLRT2 specific regulation of tight junctions and BBB permeability.

(a) mRNA fold change levels of *Flrt2* and tight junction *Claudin-5* (*Cldn5*) in bEnd.3 cells treated with control and *Flrt2* siRNA. n = 5 independent experiments. Two-tailed unpaired t-test, p = 0.023 (*Flrt2*) and 0.005 (*Cldn5*). **(b)** mRNA expression quantification of *Cldn5* in control and *Flrt2*$^{i\Delta EC}$ total brains at P7-P8. n = 6 animals per genotype. Two-tailed unpaired t-test, p = 0.009. **(c, e)** Representative immunoblots from total brain lysates of control and *Flrt2*$^{i\Delta EC}$ mice showing tight junctions proteins ZO-1 (c) and JAM-A (e) levels. **(d, f)** Quantification of protein ZO-1 (d) and JAM-A (f) levels in total brain lysates from control and *Flrt2*$^{i\Delta EC}$ mice. n = 5 animals per genotype (d), 4 control and 5 mutant animals (f **).** Two-tailed unpaired t-test, p = 0.989 (d), 0.938 (f). **(g, h)** Neocortical blood vessels stained for ZO-1 (g) and JAM-A (h) showing no differences in tight junction proteins distribution between control and Flrt2$^{i\Delta EC}$ mice. **(i)** Representative fluorescent whole-brain images of control and *Flrt2*$^{i\Delta EC}$ littermates injected with AlexaFluor555nm-conjugated Ovalbumin (45 kDa) at P7-8. **(j)** Quantification of ovalbumin whole-brain intensity in control and *Flrt2*$^{i\Delta EC}$ mice at P7-8. n = 4 animals per genotype. Two-tailed unpaired t-test, p = 0.382. **(k)** Representative fluorescent whole-brain images of control and *Flrt2*$^{i\Delta EC}$ littermates injected with AlexaFluor555nm-conjugated cadaverine at P5. **(l)** Quantification of cadaverine whole-brain intensity in control and *Flrt2*$^{i\Delta EC}$ mice at P5. n = 7 control and 8 mutant mice. Two-tailed unpaired t-test, p = 0.292. Scale bars: 10 µm (g, h), 1 mm (i, k). Data are shown as mean ± SEM. *P < 0.05, **P < 0.01, ns = not significant.

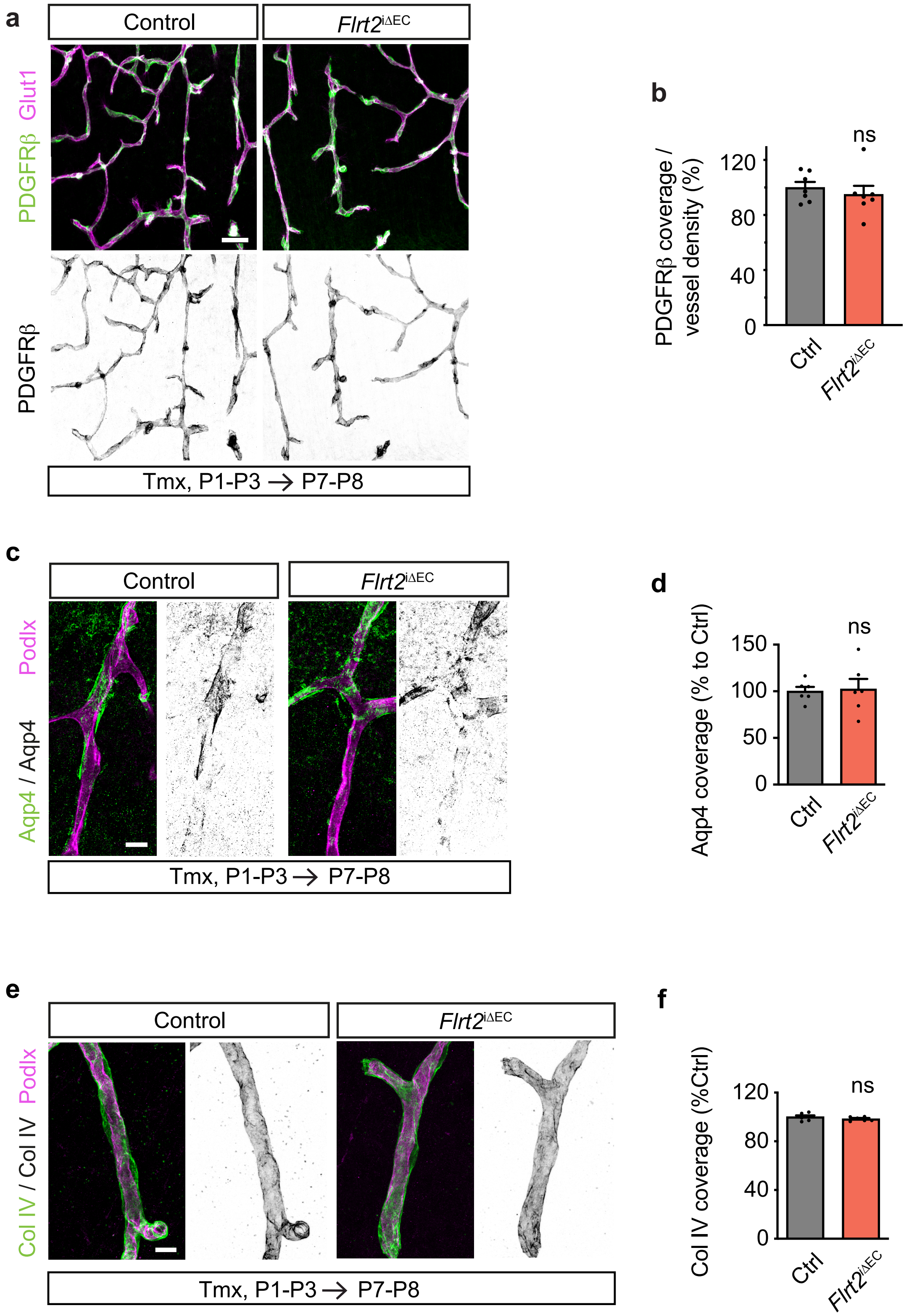
a
Control
Flrt2iΔEC
PDGFRβ Glut1
PDGFRβ
Tmx, P1-P3 → P7-P8
b
PDGFRβ coverage / vessel density (%)
120
80
40
0
ns
Ctrl
Flrt2iΔEC
c
Control
Flrt2iΔEC
Podlx
Aqp4 / Aqp4
Tmx, P1-P3 → P7-P8
d
Aqp4 coverage (% to Ctrl)
150
100
50
0
ns
Ctrl
Flrt2iΔEC
e
Control
Flrt2iΔEC
Col IV / Col IV Podlx
Tmx, P1-P3 → P7-P8
f
Col IV coverage (%Ctrl)
120
80
40
0
ns
Ctrl
Flrt2iΔEC

Supplementary Fig. 7| Other components of the neurovascular unit are not affected by the absence of endothelial FLRT2.

(a, c, e,) Cerebral cortices of control and vascular *Flrt2* mutant mice stained with the pericyte marker PDGFRβ (a), astrocytic end-feet marker Aquaporin-4 (Aqp4) (c) and extracellular matrix marker Collagen IV (Col IV) (e) and blood vessel markers Glut1 (a) or Podxl (c, e). **(b, d, f)** Quantification of PDGFRβ (b), Aqp4 (d) or Col IV (f) coverage of cortical vasculature. n = 7 (b) and 6 (d, f) animals per genotype. Two-tailed unpaired t-test, p = 0.508 (b), 0.854 (d), 0.259 (f).

Scale bars: 50 µm (a), 10 µm (c, e). Data are shown as mean ± SEM. ns = not significant.

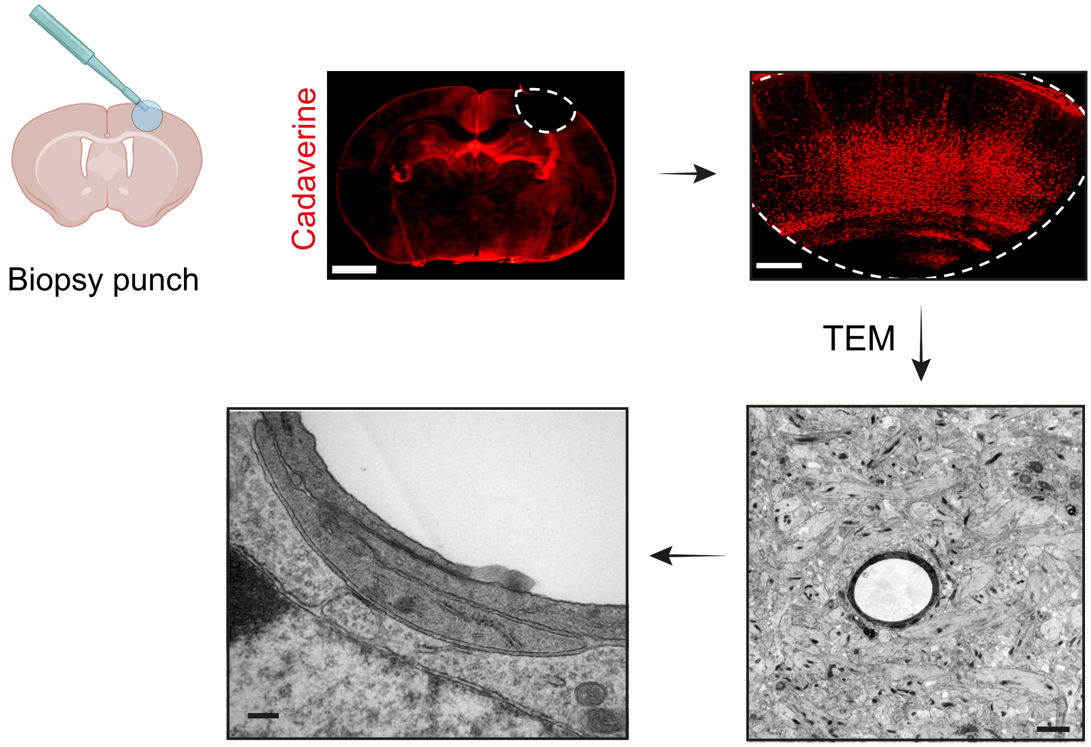
Biopsy punch
Cadaverine
TEM

Supplementary Fig. 8| TEM sample preparation.

Representative image of an endothelial-specific *Flrt2* mutant cerebral cortex section showing a leakage area where the punch was performed and further processed for transmission electron microscopy (TEM) analysis. After assessing the quality of the tissue, imaging was processed focusing in capillary EC tight junctions. Cartoon created in Biorender.com (Kirchmaier, B. (2024) BioRender.com/p09v076).

Scale bars: 1mm, 250 µm, 2500 nm, 250 nm
