# Supplementary material for: Vascular FLRT2 regulates venous-mediated angiogenic expansion and CNS barriergenesis
Source: Nat Commun. 2024 Nov 29;15:10372. doi: 10.1038/s41467-024-54570-x (PMC11604978; doi:10.1038/s41467-024-54570-x)
Supplement: Supplementary file 2 — Reporting Summary [file 41467_2024_54570_MOESM2_ESM.pdf]

nature portfolio

Corresponding author(s): Amparo Acker-Palmer

Last updated by author(s): 10.09.2024

Reporting Summary

Nature Portfolio wishes to improve the reproducibility of the work that we publish. This form provides structure for consistency and transparency in reporting. For further information on Nature Portfolio policies, see our Editorial Policies and the Editorial Policy Checklist.

Statistics

For all statistical analyses, confirm that the following items are present in the figure legend, table legend, main text, or Methods section.

n/a	Confirmed	
☐	☒	The exact sample size (*n*) for each experimental group/condition, given as a discrete number and unit of measurement
☐	☒	A statement on whether measurements were taken from distinct samples or whether the same sample was measured repeatedly
☐	☒	The statistical test(s) used AND whether they are one- or two-sided<br>*Only common tests should be described solely by name; describe more complex techniques in the Methods section.*
☒	☐	A description of all covariates tested
☐	☒	A description of any assumptions or corrections, such as tests of normality and adjustment for multiple comparisons
☐	☒	A full description of the statistical parameters including central tendency (e.g. means) or other basic estimates (e.g. regression coefficient) AND variation (e.g. standard deviation) or associated estimates of uncertainty (e.g. confidence intervals)
☐	☒	For null hypothesis testing, the test statistic (e.g. *F*, *t*, *r*) with confidence intervals, effect sizes, degrees of freedom and *P* value noted<br>*Give P values as exact values whenever suitable.*
☒	☐	For Bayesian analysis, information on the choice of priors and Markov chain Monte Carlo settings
☒	☐	For hierarchical and complex designs, identification of the appropriate level for tests and full reporting of outcomes
☒	☐	Estimates of effect sizes (e.g. Cohen's *d*, Pearson's *r*), indicating how they were calculated

Our web collection on statistics for biologists contains articles on many of the points above.

Software and code

Policy information about availability of computer code

Data collection	Immunostaining and tracer fluorescent signal detected with Leica SP5 and Leica SP8 confocal microscopes. Images of expanded cells were obtained with inverted Zeiss LSM 980 confocal microscope; Leica M205 FCA stereo microscope was used to image whole brain samples.<br>TEM imaging was performed with Zeiss microscope, type EM 109 with a 2K-CCD-Camera from TRS.<br>qPCR was performed with Stop One Plus Real-Time PCR System (Applied Biosystems)<br>western blot signal was detected with ImageQuantTM LAS 4000 system
Data analysis	Angiotool software (version 0.6) and ImageJ (National Institutes of Health)/Fiji (version 2.14/1.54f) were used for image data analysis<br>Image StudioTM Lite software (version 5.2.5) was used for Western blot quantification<br>GraphPad Prism (version 8) was used for statistical analysis

For manuscripts utilizing custom algorithms or software that are central to the research but not yet described in published literature, software must be made available to editors and reviewers. We strongly encourage code deposition in a community repository (e.g. GitHub). See the Nature Portfolio guidelines for submitting code & software for further information.

Data

Policy information about availability of data

All manuscripts must include a data availability statement. This statement should provide the following information, where applicable:

- Accession codes, unique identifiers, or web links for publicly available datasets
- A description of any restrictions on data availability
- For clinical datasets or third party data, please ensure that the statement adheres to our policy

Source data are provided with this paper as a Source Data file.

Research involving human participants, their data, or biological material

Policy information about studies with human participants or human data. See also policy information about sex, gender (identity/presentation), and sexual orientation and race, ethnicity and racism.

Reporting on sex and gender	none
Reporting on race, ethnicity, or other socially relevant groupings	none
Population characteristics	none
Recruitment	none
Ethics oversight	none

Note that full information on the approval of the study protocol must also be provided in the manuscript.

Field-specific reporting

Please select the one below that is the best fit for your research. If you are not sure, read the appropriate sections before making your selection.

☒ Life sciences ☐ Behavioural & social sciences ☐ Ecological, evolutionary & environmental sciences

For a reference copy of the document with all sections, see nature.com/documents/nr-reporting-summary-flat.pdf

Life sciences study design

All studies must disclose on these points even when the disclosure is negative.

Sample size	All animal experiments enclose at least 3 animals per genotype (unless otherwise specified), and animals were obtained from at least 2 different litters. All in vitro experiments were carried out 3 times, unless otherwise specified. Information is included in methods and figure legends.
Data exclusions	test for outliers was performed using ROUT methods from GraphPad Prism
Replication	All experiments include at least 3 biological replicates, unless otherwise specified.
Randomization	For mouse experiments, the entire postnatal litter was injected with tamoxifen and mice were equally treated. Mice were allocated into different groups based on their genotype.
Blinding	The investigators were blind at the time of experiment execution and data quantification.

Reporting for specific materials, systems and methods

We require information from authors about some types of materials, experimental systems and methods used in many studies. Here, indicate whether each material, system or method listed is relevant to your study. If you are not sure if a list item applies to your research, read the appropriate section before selecting a response.

Materials & experimental systems

n/a	Involved in the study
☐	☒ Antibodies
☐	☒ Eukaryotic cell lines
☒	☐ Palaeontology and archaeology
☐	☒ Animals and other organisms
☒	☐ Clinical data
☒	☐ Dual use research of concern
☒	☐ Plants

Methods

n/a	Involved in the study
☒	☐ ChIP-seq
☒	☐ Flow cytometry
☒	☐ MRI-based neuroimaging

Antibodies

Antibodies used

-Antibodies for immunostaining
Primary antibodies (tissue)
goat anti-podocalyxin (AF1556, R&D), mouse anti-NeuN (Millipore; MAB377), goat anti-FLRT2 (AF2877, R&D), rabbit anti-Glut1 (07-1401, Millipore), rabbit anti-collagen IV (2150-1470, Bio-rad), rabbit anti-Calpain-2 (ab39165, Abcam), rabbit anti-ERG (ab92513, Abcam), rat anti-VE-cadherin (550274, BD Pharmingen), mouse anti-GM130 (610823, BD Biosciences), rabbit anti-Numb (2756, Cell Signaling), mouse anti-Claudin 5 (35-2500, Invitrogen), rabbit anti-RCFP (632475, Takara Bio), rabbit anti-cleaved caspase-3 (9661, NEB), rat anti-p21 (ab107099, Abcam), rat anti-JAM-A (MABT128, Millipore), mouse anti-ZO-1 (3309100, Invitrogen), goat anti-PDGFRβ (GT 15065, Neuromics), and rabbit anti-Aquaporin 4 (AB2218, Millipore).
Primary antibodies (cells)
mouse anti-VE-cadherin (sc-9989, Santa Cruz), mouse anti-VE-cadherin (MABT134, Millipore), goat anti-VE-cadherin (AF1002, R&D), rabbit anti-Calpain-2 (ab39165, Abcam), rabbit anti-Calpain-1 (2556, Cell signaling) goat anti-FLRT2 (AF2877, R&D), rabbit anti-Numb (2756, Cell Signaling) and mouse anti-GM130 (610823, BD Biosciences) and rat anti-Pecam1 (550274, BD Pharmingen).
Secondary antibodies:
donkey anti-mouse/rabbit/goat/rat conjugated to Alexa Fluor -488, -555, -568, -594, and -647 (Life Technologies)

-Antibody for antibody feeding assay:
VE-cadherin antibody (clone BV6, MABT134, Merck Millipore) and VE-cadherin antibody (goat, AF1002, R&D Systems).

-Antibodies for Western blot and IP
Primary antibodies: goat anti-VE-cadherin (AF1002, R&D), goat anti-FLRT2 (AF2877, R&D), mouse anti-Numb 1 (sc-136554, Santa Cruz), rabbit anti- -catenin (8480, Cell Signaling), rabbit anti-FoxO1 (2880, Cell Signaling), mouse anti-Claudin-5 (35-2500, Invitrogen), rat anti-JAM-A (MABT128, Millipore), and rabbit anti-ZO-1 (61-7300, Invitrogen).
Loading controls: Mouse anti- -tubulin (A-11126, Invitrogen), mouse anti- -actin (sc-47778, Santa Cruz), mouse anti-pan-cadherin (C1821, Sigma), and rabbit anti-lamin A/C (2032, Cell Signaling).
HRP-conjugated secondary antibodies: donkey anti-goat (705-035-147, Jackson Immuno Research Laboratories), goat anti-mouse (115-035-146, Jackson Immuno Research Laboratories), donkey anti-rabbit (10379664, ThermoFisher) and goat anti-rat (NA935V, GE Healthcare).

-Antibodies for FISH
Anti-digoxigenin-AP (alkaline phosphatase) antibody (Fab fragments, Roche #11093274910)

Validation

All primary antibodies used in the study were commercial and used according to the profile of manufactures. All other details are provided in the methods section.

Eukaryotic cell lines

Policy information about cell lines and Sex and Gender in Research

Cell line source(s)

Huvec were obtained from Lonza (CC2519); bEnd.3 (CRL-2299) were obtained from ATCC.

Authentication

Huvec are guaranteed to express CD31 and CD105 (authentication by vendor); bEnd3 endothelial nature was confirmed by expression of von Willebrand factor, ICAM1, VCAM1, MadCAM1, and uptake of LDL (authentication made by vendor/donor).

Mycoplasma contamination

none

Commonly misidentified lines
(See ICLAC register)

none

Animals and other research organisms

Policy information about studies involving animals; ARRIVE guidelines recommended for reporting animal research, and Sex and Gender in Research

Laboratory animals

Individually ventilated cages were used to host the mice, maintaining them under specific germ-free conditions, in 12 hours day/night light cycles, temperature 22 C, humidity 55%, Aspen bedding, and with food and water ab libitum. The following mouse lines

	were used: Cdh5-creERT2 (provided by R. Adams), Flrt2lox/lox (provided by R. Klein) and RosatdTomato (Jackson lab strain 007914), wildtype C57BL/6N (Charles River). Experimental animals were analysed at postnatal day (P) 5 and P7 or P8.
Wild animals	No wild animals were used in this study.
Reporting on sex	Both female and male mice were used in the study.
Field-collected samples	The study did not involve samples collected from the field.
Ethics oversight	All animal experiments were approved by the Regierungspräsidium of Darmstadt and the Veterinäramt of Frankfurt am Main and performed under the permits V54-19 c 20/15 – FR/1016, V54-19 c 20/15 – FR/2006, and V54-19 c 20/15 – FR/2010.

Note that full information on the approval of the study protocol must also be provided in the manuscript.

Plants

Seed stocks	none
Novel plant genotypes	none
Authentication	none
